# Stabilities for Nonisentropic Euler-Poisson Equations

**DOI:** 10.1155/2015/494707

**Published:** 2015-03-16

**Authors:** Ka Luen Cheung, Sen Wong

**Affiliations:** Department of Mathematics and Information Technology, The Hong Kong Institute of Education, 10 Lo Ping Road, Tai Po, New Territories, Hong Kong

## Abstract

We establish the stabilities and blowup results for the nonisentropic Euler-Poisson equations by the energy method. By analysing the second inertia, we show that the classical solutions of the system with attractive forces blow up in finite time in some special dimensions when the energy is negative. Moreover, we obtain the stabilities results for the system in the cases of attractive and repulsive forces.

## 1. Introduction

The compressible nonisentropic Euler (*δ* = 0) or Euler-Poisson (*δ* = ±1) system for fluids can be written as

(1) $$\begin{array}{c} \rho_{t}+\nabla \cdot \left(\rho u\right)=0,\\ {\left(\rho u\right)}_{t}+\nabla \cdot \left(\rho u⊗u\right)+\nabla P+\beta \rho u=-\delta \rho \nabla \Phi ,\\ S_{t}+u\cdot \nabla S=0,\\ \Delta \Phi \left(t,x\right)=\alpha \left(N\right)\rho ,\\ P=K\rho^{\gamma }e^{S}, \end{array}$$

where *β* ≥ 0 is the frictional damping constant and *α*(*N*) is a constant related to the unit ball in ℝ^*N*^. *α*(1) = 2, *α*(2) = 2*π* and

(2) $$\begin{array}{c} \alpha \left(N\right)=N\left(N-2\right)V\left(N\right), \end{array}$$

where *V*(*N*) is the volume of the unit ball in ℝ^*N*^. As usual, *ρ* = *ρ*(*t*, *x*) ≥ 0, *u* = *u*(*t*, *x*) ∈ ℝ^*N*^, and *S*(*t*, *x*) are the density, the velocity, and the entropy, respectively. *P* is the pressure function, for which the constants *K* ≥ 0 and *γ* ≥ 1.

When *δ* = 1, the system is self-attractive. The system (1) is the Newtonian description of gaseous stars [1]. When *δ* = −1, the system comprises the Euler-Poisson equations with repulsive forces and can be used as a semiconductor model [2, 3]. When *δ* = 0, the system comprises the compressible Euler equations and can be applied as a classical model in fluid mechanics [3]. For more classical and recent results in these systems, readers can refer to [1, 4–10].

It is well known that the solution for the Poisson equation (1)_4_ can be written as

(3) $$\begin{array}{c} \Phi \left(t,x\right)=\alpha \left(N\right)\int_{R^{N}}G\left(x-y\right)\rho \left(t,y\right)dy, \end{array}$$

where *G* is the Green's function for the Poisson equation in the *N*-dimensional spaces defined by

(4) $$\begin{array}{c} G\left(x\right):=\left\{\begin{array}{cc} \left|x\right|, & N=1;\\ \log\left|x\right|, & N=2;\\ \frac{-1}{{\left|x\right|}^{N-2}}, & N\geq 3. \end{array}\right. \end{array}$$

*Notation*. In the following discussion, classical solutions (*ρ*, *u*, *S*) are *C* ^1^ solutions with compact support *Ω* = *Ω*(*t*) for each fixed time *t*. We also denote the total mass by *M*, where

(5) $$\begin{array}{c} M=\int_{\Omega }\rho \;dx=\int_{\Omega (0)}\rho_{0}\;dx, \end{array}$$

where *ρ*_0_ = *ρ*_0_(*x*): = *ρ*(0, *x*).

Lastly, we will denote

(6) $$\begin{array}{c} R\left(t\right)=\text{the}\;\text{diameter}\;\text{of}\;\Omega \left(t\right). \end{array}$$

## 2. Lemmas

In this section, we establish some lemmas for the proof of the main results. The following lemma will be used to derive the energy functional for *γ* > 1; namely,

(7) $$\begin{array}{c} E\left(t\right)=\int_{\Omega }\left(\frac{1}{2}\rho {\left|u\right|}^{2}+\frac{1}{\gamma -1}P\right)dx+\frac{\delta }{2}\int_{\Omega }\rho \Phi \;dx \end{array}$$

is conserved in time if the system (1) is not damped.

**Lemma 1 .**

For the classical solution (*ρ*, *u*, *S*) of system (1), we have

(8) $$\begin{array}{c} \int_{\Omega }P_{t}dx=\left(\gamma -1\right)\int_{\Omega }u\cdot \nabla P\;dx, \end{array}$$

where *P* is defined by (1)_*5*_.

**Proof**

We have

(9) Pt=KργeSSt+eSγργ−1ρt by  15=PSt+KeSγργ−1−∇·ρu by  15  and  11=P−u·∇S+KeSγργ−1−∇·ρu by  13=−P∑i=1Nui∂iS−KγeSργ−1∑i=1Nρ∂iui+ui∂iρ=−γP∑i=1N∂iui−∑i=1NP∂iS+KγeSργ−1∂iρui by  15=−γP∑i=1N∂iui−∑i=1N∂iPui=−γP∇·u−u·∇P.

Note that, by Divergence Theorem,

(10) $$\begin{array}{c} -\int_{\Omega }P\nabla \cdot u\;dx=\int_{\Omega }u\cdot \nabla P\;dx. \end{array}$$

Thus,

(11) $$\begin{array}{c} \int_{\Omega }P_{t}\;dx=\left(\gamma -1\right)\int_{\Omega }u\cdot \nabla P\;dx. \end{array}$$

Next, the results of the following two lemmas will be used in the derivations of the energy functionals for both *γ* > 1 and *γ* = 1. It will be shown that in Section 3 the energy functional for *γ* = 1 is

(12) $$\begin{array}{c} E\left(t\right)=\int_{\Omega }\left(\frac{1}{2}\rho {\left|u\right|}^{2}+K\rho e^{S}\left(\ln\rho -1\right)\right)dx+\frac{\delta }{2}\int_{\Omega }\rho \Phi \;dx \end{array}$$

which is conversed in time if the system (1) is not damped.

**Lemma 2 .**

For the classical solution (*ρ*, *u*, *S*) of system (1), we have

(13) ∫Ω12ρu2tdx=−∫Ωu·∇P−δ∫Ω(∇Φ)·(ρu)dx −β∫Ωρu2dx,

where *P* is defined by (1)_*5*_ and Φ is the solution of (1)_*4*_.

**Proof**

We have

(14)  12ρu2t

(15)    =ρut·u−12ρtu2 (by  product  rule)

(16)     =−δρ∇Φ+∇·ρu⊗u+∇P+βρu·u

One can check a detail proof of the following equality in the Appendix:

(17) $$\begin{array}{c} \int_{\Omega }\left(\frac{1}{2}{\left|u\right|}^{2}\nabla \cdot \left(\rho u\right)-u\cdot \left[\nabla \cdot \left(\rho u⊗u\right)\right]\right)dx=0. \end{array}$$

Thus,

(18) $$\begin{array}{c} \int_{\Omega }{\left(\frac{1}{2}\rho {\left|u\right|}^{2}\right)}_{t}dx=-\int_{\Omega }\left(\delta \rho \nabla \Phi +\nabla P+\beta \rho u\right)\cdot u\;dx \end{array}$$

by (16) and (17).

Thus, the proof is complete.

**Lemma 3 .**

For the classical solution (*ρ*, *u*, *S*) of system (1), we have

(19) $$\begin{array}{c} \int_{\Omega }\left(\nabla \Phi \right)\cdot \left(\rho u\right)dx=\int_{\Omega }\Phi \rho_{t}dx=\frac{1}{2}\int_{\Omega }{\left[\rho \Phi \right]}_{t}dx, \end{array}$$

where Φ is the solution of (1)_*4*_.

**Proof**

We have

(20) ∫Ω∇Φ·ρudx  =−∫ΩΦ∇·ρudx by  Divergence  Theorem  =∫ΩΦρtdx by  11.

Thus, the first equality in (19) holds.

Next,

(21) ∫ΩΦρtdx=1αN∫ΩΦΔΦtdx by  14=1αN∫ΩΔΦΦtdx by  Green's  Formula=∫ΩρΦtdx by  14.

Thus, the second equality in (19) holds.

The lemma below is crucial to obtaining the energy functional for *γ* = 1. Comparing the left hand sides of (8) and (22), we note that the left hand side of (22) (given in the next lemma), which contains the term ln⁡*ρ* − 1, is nontrivial to be found.

**Lemma 4 .**

For the classical solution (*ρ*, *u*, *S*) of system (1) with *γ* = 1, we have

(22) $$\begin{array}{c} \frac{\text{d}}{\text{d}t}\int_{\Omega }K\rho e^{S}\left(\ln\rho -1\right)dx=\int_{\Omega }u\cdot \nabla P\;dx, \end{array}$$

where *P* is defined by (1)_*5*_.

**Proof**

Note that

(23) ρeSln⁡ρ−1t  =eSρln⁡ρ−1t+ρln⁡ρ−1eSSt  =eSρt+ln⁡ρ−1ρt+eSρln⁡ρ−1St  =eS(ln⁡ρ)ρt+eSρ(ln⁡ρ−1)St  =−eSln⁡ρ∇·ρu−eSρln⁡ρ−1u·∇Smmmmmmmmmmmmmmmmmby  11  and  13.

Thus,

(24) ddt∫ΩKρeS(ln⁡ρ−1)dx  =−∫ΩKeSln⁡ρ∇·ρudx   −∫ΩKeSρln⁡ρ−1u·∇Sdx  =∫Ω(ρu)·∇KeSln⁡ρdx   −∫ΩKeSρln⁡ρ−1u·∇Sdxmmmmmmlmby  Divergence  Theorem  =∫Ωu·Kρ∇eSln⁡ρ−KeSρln⁡ρ−1∇Sdx  =∫Ωu·KeS∇ρ+KeSρ∇Sdx  =∫Ωu·∇P dx by  15.

The proof is complete.

## 3. Main Results

In this section, we find out the energy functionals for the system (1) in the case of *γ* > 1 (Proposition 5) and *γ* = 1 (Proposition 6). Moreover, we establish the stabilities results (Proposition 8) and a blowup result (Proposition 9) for system (1).

**Proposition 5 .**

For the classical solution (*ρ*, *u*, *S*) of system (1) with *γ* > 1, let

(25) $$\begin{array}{c} E\left(t\right)=\int_{\Omega }\left(\frac{1}{2}\rho {\left|u\right|}^{2}+\frac{1}{\gamma -1}P\right)dx+\frac{\delta }{2}\int_{\Omega }\rho \Phi \;dx. \end{array}$$

Then,

(26) $$\begin{array}{c} \dot{E}\left(t\right)=-\beta \int_{\Omega }\rho {\left|u\right|}^{2}dx, \end{array}$$

where $\dot{E}(t)$ is the devertive of *E*(*t*) with respect to *t*.

Thus, *E*(*t*) is a decreasing function and is conserved if the system is not damped.

**Proof**

By Lemma 1,

(27) $$\begin{array}{c} \frac{1}{\gamma -1}\int_{\Omega }P_{t}dx=\int_{\Omega }u\cdot \nabla P\;dx. \end{array}$$

By Lemma 2,

(28) ∫Ω12ρu2tdx=−∫Ωu·∇P−δ∫Ω(∇Φ)·(ρu)dx −β∫Ωρu2dx.

By Lemma 3,

(29) $$\begin{array}{c} \int_{\Omega }\left(\nabla \Phi \right)\cdot \left(\rho u\right)dx=\frac{1}{2}\int_{\Omega }{\left[\rho \Phi \right]}_{t}dx. \end{array}$$

Thus, the proof is complete.

**Proposition 6 .**

For the classical solution (*ρ*, *u*, *S*) of system (1) with *γ* = 1, let

(30) $$\begin{array}{c} E\left(t\right)=\int_{\Omega }\left(\frac{1}{2}\rho {\left|u\right|}^{2}+K\rho e^{S}\left(\ln\rho -1\right)\right)dx+\frac{\delta }{2}\int_{\Omega }\rho \Phi \;dx. \end{array}$$

Then,

(31) $$\begin{array}{c} \dot{E}\left(t\right)=-\beta \int_{\Omega }\rho {\left|u\right|}^{2}dx, \end{array}$$

where $\dot{E}(t)$ is the derivative of *E*(*t*) with respect to *t*.

Thus, *E*(*t*) is a decreasing function and is conserved if the system is not damped.

**Proof**

By Lemma 2,

(32) ∫Ω12ρu2tdx=−∫Ωu·∇P−δ∫Ω(∇Φ)·(ρu)dx −β∫Ωρu2dx.

By Lemma 3,

(33) $$\begin{array}{c} \int_{\Omega }\left(\nabla \Phi \right)\cdot \left(\rho u\right)dx=\frac{1}{2}\int_{\Omega }{\left[\rho \Phi \right]}_{t}dx. \end{array}$$

By Lemma 4,

(34) $$\begin{array}{c} \frac{\text{d}}{\text{d}t}\int_{\Omega }K\rho e^{S}\left(\ln\rho -1\right)dx=\int_{\Omega }u\cdot \nabla P\;dx. \end{array}$$

Thus, the proof is complete.

**Proposition 7 .**

Let

(35) $$\begin{array}{c} H\left(t\right)=\int_{\Omega }\rho {\left|x\right|}^{2}dx. \end{array}$$

We have

(36) H¨t=−βH˙t+2∫Ωρu2+2Pdx−2πδM2,mmmmmmmmmmmmmmmmmfor  N=2,H¨t=−βH˙(t)+2∫Ωρu2+NPdx +N−2δ∫ΩρΦ dx, for  N≥3,

where $\dot{H}(t)=(\text{d}/\text{d}t)H(t)$, $\ddot{H}(t)=(\text{d}/\text{d}t)\dot{H}(t)$, (*ρ*, *u*, *S*) is a classical solution of system (1), and *M* is defined by (5).

**Proof**

(37) H˙t=−∫Ω∇·(ρu)x2dx by  11=∫Ω∇x2·ρudx by  Divergence  Theorem=∫Ω2x·ρudx,H¨t=2∫Ωx·−∇·ρu⊗u−∇P−δρ∇Φ−βρudxmmmmmmmmmmmmmmmmmmmmmby  12=−βH˙(t)+2∫Ωx·−∇·ρu⊗u−∇P−δρ∇Φdx.

We split the last term of the above equality into three parts.

Firstly,

(38) −∫Ωx·∇·ρu⊗udx  =−∑j=1N∫Ωxj∑i=1N∂iρuiujdx by  definitions  =∫Ωρu2dx. by  integration  by  parts.

Secondly,

(39) −∫Ωx·∇P dx=∫ΩP∇·x dx by  Divergence  Theorem=∫ΩNP dx.

Thirdly,

(40) −δ∫Ωρx·∇Φ dx  =−δαN∫Ω∫Ωρxρy∇xGx−y·xdy dxmmmmmmmmmmmmmmmmmmmmmmmmmby  (3)  =:−δα(N)I,

where ∇_*x*_ is the gradient operator with respect to the spatial variable *x*.

Note that

(41) I=∫Ω∫Ωρxρy∇xGx−y·xdy dx=∫Ω∫Ωρ(x)ρ(y)[∇xG(x−y)·(x−y)]dy dx +∫Ω∫Ωρxρy∇xGx−y·ydy dx.

For *N* = 2,

(42) $$\begin{array}{c} \nabla_{x}G\left(x\right)=\nabla_{x}\log\left|x\right|=\frac{1}{{\left|x\right|}^{2}}x. \end{array}$$

Thus,

(43) I=∫Ω∫Ωρxρydy dx−I,I=12∫Ω∫Ωρ(x)ρ(y)dy dx=M22.

The result for *N* = 2 is established.

For *N* ≥ 3,

(44) $$\begin{array}{c} \nabla_{x}G\left(x\right)=-\nabla_{x}\frac{1}{{\left|x\right|}^{N-2}}=\left(N-2\right)\frac{1}{{\left|x\right|}^{N}}x. \end{array}$$

Thus,

(45) I=∫Ω∫Ωρxρy∇xGx−y·x−ydy dx +∫Ω∫Ωρ(x)ρ(y)[∇xG(x−y)·y]dy dx=−N−2αN∫ΩρΦ dx−I,I=−N−22α(N)∫ΩρΦ dx.

The results for *N* ≥ 3 are also established.

Now, we are ready to present the stability results.

**Proposition 8 .**

Considering the classical solutions of system (1), we have the following.

*Case 1.* For *δ* = 1, *β* = 0, *N* = 3 or 4, *γ* ≥ 2(*N* − 1)/*N*, and *E*(0) ≥ 0, we have

(46) $$\begin{array}{c} \underset{t\to \infty }{\lim}\inf\frac{R\left(t\right)}{t}\geq \sqrt{\frac{\left(N-2\right)E\left(0\right)}{M}}. \end{array}$$

*Case 2.* For *δ* = −1, *β* = 0, *N* ≥ 4, and *γ* ≥ (*N* + 2)/*N*, we have

(47) $$\begin{array}{c} \underset{n\to \infty }{\lim}\;\inf\frac{R\left(t\right)}{t}\geq \sqrt{\frac{2E\left(0\right)}{M}}. \end{array}$$

*Case 3.* For *δ* = −1, *β* = 0, *N* = 2, and *γ* > 1, we have

(48) $$\begin{array}{c} \underset{n\to \infty }{\lim}\inf\frac{R\left(t\right)}{t}\geq \sqrt{\pi M}. \end{array}$$

**Proof**

First of all, by definitions (35), (6), and (5), we always have

(49) $$\begin{array}{c} H\left(t\right)=\int_{\Omega (t)}\rho {\left|x\right|}^{2}dx\leq R^{2}\left(t\right)M. \end{array}$$

*Case 1.* By Propositions 5 and 7, we have

(50) H¨t=2∫Ωρu2+NPdx+(N−2)∫ΩρΦ dx=2∫Ωρu2dx+2N∫ΩP dx +(N−2)2E(t)−2γ−1∫ΩP dx−∫Ωρu2dx=2−(N−2)∫Ωρu2dx +2N−2(N−2)γ−1∫ΩP dx+2(N−2)E(t)≥2(N−2)E(t)=2(N−2)E(0).

Thus,

(51) $$\begin{array}{c} H\left(t\right)\geq \left(N-2\right)E\left(0\right)t^{2}+\dot{H}\left(0\right)t+H\left(0\right). \end{array}$$

In view of inequality (49), we have

(52) $$\begin{array}{c} \frac{R\left(t\right)}{t}\geq \sqrt{\frac{\left(N-2\right)E\left(0\right)}{M}+\frac{\dot{H}\left(0\right)}{Mt}+\frac{H\left(0\right)}{Mt^{2}}}. \end{array}$$

Thus,

(53) $$\begin{array}{c} \underset{t\to \infty }{\lim}\inf\frac{R\left(t\right)}{t}\geq \sqrt{\frac{\left(N-2\right)E\left(0\right)}{M}}. \end{array}$$

*Case 2.* By Propositions 5 and 7, we have

(54) H¨t=2∫Ωρu2+NPdx+N−2∫Ωρ−Φdx=22E(t)−2γ−1∫ΩP dx−∫Ωρ(−Φ)dx +2N∫ΩP dx+(N−2)∫Ωρ(−Φ)dx≥4E(t)=4E(0).

Note that −Φ is a positive function for *N* ≥ 3 by (3) and (4). Thus,

(55) $$\begin{array}{c} H\left(t\right)\geq 2E\left(0\right)t^{2}+\dot{H}\left(0\right)t+H\left(0\right). \end{array}$$

It follows that

(56) $$\begin{array}{c} \underset{n\to \infty }{\lim}\inf\frac{R\left(t\right)}{t}\geq \sqrt{\frac{2E\left(0\right)}{M}}. \end{array}$$

*Case 3.* By Proposition 7, we have

(57) H¨t=2∫Ωρu2+2Pdx+2πM2≥2πM2.

It follows that

(58) $$\begin{array}{c} \underset{n\to \infty }{\lim}\inf\frac{R\left(t\right)}{t}\geq \sqrt{\pi M}. \end{array}$$

Finally, we can give the blowup result.

**Proposition 9 .**

If *δ* = 1, *N* ≥ 4, 1 < *γ* ≤ 2(*N* − 1)/*N*, and *E*(0) < 0, then the classical solutions of (1) blow up in finite time.

**Proof**

   

*Case 1 (β* = 0). As before, we have, from Propositions 5 and 7, that

(59) H¨t=4−N∫Ωρu2dx+2N−2N−2γ−1∫ΩP dx +2(N−2)E(t)≤0+0+2(N−2)E(t)=2(N−2)E(0).

It follows that

(60) $$\begin{array}{c} H\left(t\right)\leq \left(N-2\right)E\left(0\right)t^{2}+\dot{H}\left(0\right)t+H\left(0\right). \end{array}$$

Suppose the solutions exist globally; then for sufficient large *t*, we see that *H*(*t*) is negative as the leading coefficient of the right hand side of (60) is negative. However, *H*(*t*) is nonnegative by definition (35). This is a contradiction. As a result, the solutions blow up in finite time.

*Case 2 (β* ≠ 0). Now (59) becomes

(61) $$\begin{array}{c} \ddot{H}\left(t\right)+\beta \dot{H}\left(t\right)\leq 2\left(N-2\right)E\left(0\right). \end{array}$$

It follows by multipying an integral factor *e* ^*βt*^ on both sides and taking integration that

(62) $$\begin{array}{c} H\left(t\right)\leq A_{1}+A_{2}e^{-\beta t}+\frac{2\left(N-2\right)E\left(0\right)}{\beta }t \end{array}$$

for some constants *A*_1_ and *A*_2_. Note that this implies that *H*(*t*) is negative for sufficient large *t* as *β* > 0 and (*N* − 2)*E*(0) < 0. Therefore, the solutions blow up in finite time.

## Appendix

We here complement the proof of Lemma 2 by proving the equality (17); namely,

(A.1) $$\begin{array}{c} \int_{\Omega }\left(\frac{1}{2}{\left|u\right|}^{2}\nabla \cdot \left(\rho u\right)-u\cdot \left[\nabla \cdot \left(\rho u⊗u\right)\right]\right)dx=0. \end{array}$$

Proof Firstly, by divergence theorem,

(A.2) $$\begin{array}{c} \int_{\Omega }\frac{1}{2}{\left|u\right|}^{2}\nabla \cdot \left(\rho u\right)dx=-\frac{1}{2}\int_{\Omega }\left(\rho u\right)\cdot \nabla {\left|u\right|}^{2}dx. \end{array}$$

Secondly, by definitions of the operations,

(A.3) u·∇·ρu⊗u=∑j=1Nuj∑i=1N∂iρuiuj=∑j=1Nuj∑i=1Nuj∂i(ρui)+ρui∂iuj=∑j=1Nuj2∑i=1N∂iρui+∑j=1Nuj∑i=1Nρui∂iuj=u2∇·(ρu)+∑i=1Nρui∑j=1N12∂iuj2=u2∇·ρu+12∑i=1Nρui∂iu2=u2∇·ρu+12ρu·∇u2.

Thus,

(A.4) ∫Ωu·[∇·(ρu⊗u)]dx  =∫Ωu2∇·(ρu)+12(ρu)·∇u2dx  =∫Ω12u2∇·ρudx by  A.2.

Thus, equality (A.1) is established.
